# Impact of Cigarette Smoke on the Human and Mouse Lungs: A Gene-Expression Comparison Study

**DOI:** 10.1371/journal.pone.0092498

**Published:** 2014-03-24

**Authors:** Mathieu C. Morissette, Maxime Lamontagne, Jean-Christophe Bérubé, Gordon Gaschler, Andrew Williams, Carole Yauk, Christian Couture, Michel Laviolette, James C. Hogg, Wim Timens, Sabina Halappanavar, Martin R. Stampfli, Yohan Bossé

**Affiliations:** 1 Department of Pathology and Molecular Medicine, McMaster University, Hamilton, Ontario, Canada; 2 Centre de Recherche de l’Institut universitaire de cardiologie et de pneumologie de Québec, Université Laval, Quebec city, Québec, Canada; 3 Center for Heart and Lung Health St. Paul’s Hospital, University of British Columbia, Vancouver, British Columbia, Canada; 4 Department of Medicine Respiratory Division, University of British Columbia, Vancouver, British Columbia, Canada; 5 Department of Pathology and Medical Biology University Medical Center Groningen, University of Groningen, Groningen, Netherlands; 6 Environmental and Radiation Health Sciences Directorate, Health Canada, Ottawa, Ontario, Canada; 7 Department of Medicine Firestone Institute of Respiratory Health at St. Joseph’s Healthcare, McMaster University, Hamilton, Ontario, Canada; 8 Department of Molecular Medicine, Laval University, Quebec city, Québec, Canada; University of Bern, Switzerland

## Abstract

Cigarette smoke is well known for its adverse effects on human health, especially on the lungs. Basic research is essential to identify the mechanisms involved in the development of cigarette smoke-related diseases, but translation of new findings from pre-clinical models to the clinic remains difficult. In the present study, we aimed at comparing the gene expression signature between the lungs of human smokers and mice exposed to cigarette smoke to identify the similarities and differences. Using human and mouse whole-genome gene expression arrays, changes in gene expression, signaling pathways and biological functions were assessed. We found that genes significantly modulated by cigarette smoke in humans were enriched for genes modulated by cigarette smoke in mice, suggesting a similar response of both species. Sixteen smoking-induced genes were in common between humans and mice including six newly reported to be modulated by cigarette smoke. In addition, we identified a new conserved pulmonary response to cigarette smoke in the induction of phospholipid metabolism/degradation pathways. Finally, the majority of biological functions modulated by cigarette smoke in humans were also affected in mice. Altogether, the present study provides information on similarities and differences in lung gene expression response to cigarette smoke that exist between human and mouse. Our results foster the idea that animal models should be used to study the involvement of pathways rather than single genes in human diseases.

## Introduction

There are more than one billion cigarette smokers on the planet; cigarette smoking being responsible for five million deaths every year worldwide [1]. Moreover, the chronic and insidious nature of smoking-related diseases can reduce the quality of life for many decades. In an effort to limit the social and economic impact of cigarette smoke on our society, it is critical to dissect and decipher the mechanisms by which cigarette smoke impacts lung biology and leads to pulmonary and systemic diseases.

In the past decades, our understanding of the cellular and molecular consequences of cigarette smoke exposure on the lung has expanded tremendously owing to new research tools, and increased availability of clinical samples and animal models. Despite major technological advancements, one important problem remains; observations made in animal models do not always reflect human pathobiology. As a consequence, we are still facing the translational challenge associated with the use of animals to model human diseases, including chronic obstructive pulmonary disease (COPD) [2].

There is currently no large-scale study comparing the pulmonary gene expression profile associated to cigarette smoke exposure in humans and mice. Such study has the potential to identify a conserved response to cigarette smoke between the two species, identify new genes and cellular pathways affected by cigarette smoke, validate the use of animal models in investigating the role played by given genes or pathways, and facilitate the translation of knowledge acquired in animal models to clinically relevant findings.

Using a human and a mouse whole-genome expression study, we identified the pulmonary genes, pathways and biological functions affected by cigarette smoke in a mouse model of cigarette smoke exposure and validated the findings in a previously described large-scale transcriptomic human data set [3]. We found that genes associated with cigarette smoke in humans were significantly enriched in the lung gene expression response to cigarette smoke in mice. We also identified 6 new genes induced by cigarette smoke in both humans and mice. In addition, we found a new conserved pulmonary response to cigarette smoke in the induction of phospholipid metabolism/degradation pathways. Finally, biological functions altered by smoking were very similar between both species.

## Methods

### Ethics Statement

Sample collection from human subjects was reviewed and approved by the *Institut universitaire de cardiologie et de pneumologie de Québec* (IUCPQ) ethics board. All subject provided written informed consent. Animal experiments were performed in respect to the Canadian Council on Animal Care policies and guidelines. McMaster’s Animal Research Ethics Board (AREB) reviewed and approved animal experimentation protocols.

### Human Data Set Characteristics

Human subjects and the lung specimen collection were described previously [3]. Non-tumor lung specimens were collected from patients undergoing lung cancer surgery at the IUCPQ. Non-neoplastic pulmonary parenchyma was harvested from a site as distant as possible from the tumor, immediately snap-frozen in liquid nitrogen, and stored at −80°C until further processing. Lung specimens were stored at the Respiratory Health Network Biobank of the Fonds de recherche du Québec - Santé (FRQS) (www.tissuebank.ca). Clinical characteristics of subjects are shown in Table 1. Smoking status was self-reported and validated by quantification of plasma cotinine levels using high-performance liquid chromatography-tandem mass spectrometry (ACQUITY UPLC System and the Quattro Premier XE; Waters). Current smokers with cotinine levels lowered than 15 ng/mL and never smokers with cotinine levels above 0.4 ng/mL were excluded from the analysis.

**Table 1:** Clinical characteristics of subjects.

Variables	*Never smokers (n = 43)*	*Current smokers (n = 90)*
**Gender (male:female)**	13∶30 (30.2% male)	46∶44 (51.1% male)
**Age (y)**	56.6+/−11.8 (0)	62.5+/−9.5 (0)
**BMI (kg/m2)**	27.9+/−6.4 (0)	25.9+/−4.6 (2)
**FEV1% predicted**	91.3+/−15.4 (4)	75.1+/−15.5 (6)
**FVC% predicted**	95.5+/−15.2 (6)	87.7+/−14.1 (0)
**Cardiac diseases**	9 (20.9%; 0)	26 (28.9%; 0)
**Diabetes**	2 (4.7%; 0)	11 (12.2%; 0)
**COPD**	7 (19.4; 7)	60 (71.4%; 6)
**Asthma**	1 (2.3%; 0)	2 (2.2%; 0)

**Note:** Continuous variables are mean+/−SD. The number of missing values is shown between parentheses.

### Animals, Cigarette Smoke Exposure and Lung Tissue Processing

Female BALB/c mice were obtained from Charles River at 6–8 weeks of age. Using a whole body exposure system (SIU48, PROMECH LAB AB, Vintrie, Sweden), mice (5 per group) were exposed to room air or the mainstream cigarette smoke of twelve 3R4F reference cigarettes (University of Kentucky, Lexington, USA) with filters removed 5 days per week, twice daily for 50 minutes/exposure for 8 weeks, as previously described [4]–[6]. Control animals were exposed to room air only. Lungs were collected and stored in RNALater at −80°C prior to RNA extraction.

### Comparison of Gene Expression and Statistical Analysis

The human and mouse genomic datasets were deposited in the GEO repository with accession number GSE23546 and GSE55127, respectively. All gene expression analyses were carried out with the R statistical software and packages (Mouse:LIMMA, Human: Affy & MASS). Human gene expression was analyzed as described previously [3]. Briefly, gene expression profiling was performed using a custom Affymetrix array (see GEO platform GLP10379). Expression values were extracted using Robust Multichip Average (RMA), adjusted for age, sex, and height, and compared between never and current smokers using Wilcoxon tests. Mice gene expression profiling was measured using the whole mouse gene expression 44K V2 microarray (Agilent Technologies) and normalized using the *limma* package. Wilcoxon tests were used to detect gene differentially expressed between mice exposed and non-exposed to smoking. Genes were considered differentially expressed for Benjamini-Hochberg corrected p-value <0.05. The lists of genes differentially expressed in human and mouse were then compared. A head-to-head comparison was performed considering the interspecies differences in gene names using the Biomart software (www.ensembl.org/biomart/martview).

Biological pathways analyses were performed using the Ingenuity Pathways Analysis software (IPA, Ingenuity Systems, www.ingenuity.com). Independent core analyses were performed in mice and humans to identify biological functions and canonical pathways enriched for smoking-induced genes. The Gene Set Enrichment Analysis (GSEA) program [7] was also used to identify gene sets from the Molecular Signatures Database (MSigDB) that were enriched with smoking-induced genes. A specific gene set was also derived from our previous study on the impact of smoking on gene expression in human lung [3]. In the later study, 3,223 transcripts were deemed significantly associated with smoking. This smoking-induced gene set was tested for enrichment against the ranked gene list from the mouse microarray experiment. All parameters in the GSEA analysis were kept as default.

## Results

### Similarities in the Number of Genes, Pathways and Biological Functions Affected by Cigarette Smoke in Humans and Mice

Humans and mice share most of their protein-coding genes, but mouse- and human-specific genes are also found [8]. We therefore initiated the analysis by identifying the number of genes in common on both gene expression microarray platforms used in this study. Out of the 20,025 genes on the human microarray and the 28,038 genes on the mouse microarray, 11,732 were interrogated on both platforms based on common gene names (Figure S1 in File S1). We then performed a “Gene Set Enrichment Analysis (GSEA)” to assess whether the genes significantly modulated by cigarette smoke in humans were enriched among the genes significantly modulated by smoking in mice. The gene set enrichment was significant (p<0.006) and suggested that, at large scale similar genes are modulated by cigarette smoke in humans and mice (Figure S2 in File S1).

**Figure 1:**
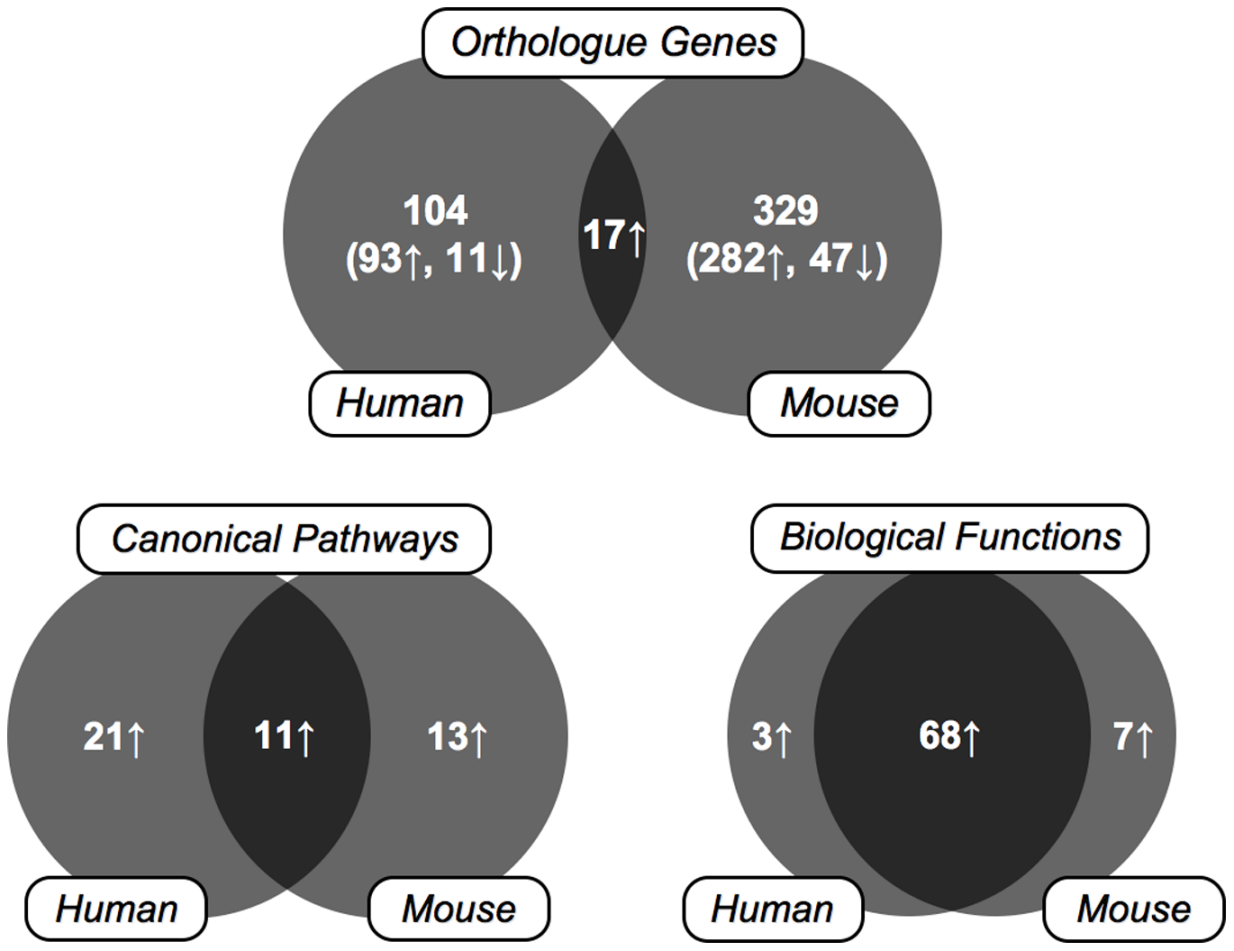
Venn diagram representing the number of orthologue genes, canonical pathways and biological functions induced by cigarette smoke and shared between humans and mice.

We then investigated the genes modulated by cigarette smoke in humans and mice in an effort to find the similarities. Out of the 3,223 human and 1,713 mouse probesets significantly modulated by cigarette smoke, 121 human and 346 mouse genes had a fold change >2, among which 17 genes were in common (*14% [human] and 4.9% [mouse]*) (Table 2). Ingenuity Pathway Analysis Software recognized 111 human genes and 294 mouse focus genes with a fold change >2 and a p-value <0.05. The gene expression signature suggested the activation of 32 and 24 pathways in the human and mouse lungs exposed to cigarette smoke, respectively. From these pathways, 11 (*34% [human] and 46% [mouse]*) were found in both species. Finally, 71 and 75 biological functions were affected by cigarette smoke in the humans and mice, respectively, 68 found in common (*96% [human] and 91% [mouse]*). These data suggest that the similarities of the effect of cigarette smoke on the lung between humans and mice are mainly found at the pathway and biological function levels rather than the single gene level (Figure 1).

**Table 2:** Genes significantly upregulated by cigarette smoke exposure in the human and mouse lungs.

		HUMANS		MICE	
Gene name	GeneAbbreviation	FoldIncrease Human	*p*-value	FoldIncrease Mice	*p*-value
Acid phosphatase 5, tartrate resistant	*ACP5*	2.2	1.46E-015	2.5	6.83E-004
Aryl hydrocarbon receptor repressor	*AHRR*	6.1	3.28E-020	11.8	1.43E-004
Aldehyde dehydrogenase 3 family, member A1	*ALDH3A1*	4.0	7.11E-015	11.2	1.05E-003
ATPase, H+ transporting, lysosomal 38kDa, V0 subunit d2	*ATP6V0D2*	5.9	7.64E-016	4.5	2.87E-003
Basic helix-loop-helix family, member e41	*BHLHE41*	2.1	1.02E-015	3.5	5.92E-004
C-type lectin domain family 5 member A	*CLEC5A*	3.3	9.15E-018	5.8	8.34E-003
Cytochrome P450 1B1	*CYP1B1*	4.5	1.14E-019	12.5	6.78E-003
Growth differentiation factor 15	*GDF15*	2.8	1.83E-014	2.3	1.06E-004
Glutathione S-Transferase Alpha 2	*GSTA2*	3.6	3.74E-006	2.18	2.78E-003
Lipocalin-2	*LCN2*	2.2	1.56E-008	6.1	2.77E-002
Matrix metalloproteinase-12	*MMP12*	4.0	1.89E-013	26.3	4.63E-003
NIMA (never in mitosis gene a)-related kinase 6	*NEK6*	2.0	1.27E-016	2.1	2.38E-003
NAD(P)H dehydrogenase, quinone 1	*NQO1*	2.0	3.24E-016	5.1	2.89E-006
Phospholipase A2, group VII	*PLA2G7*	3.4	3.82E-012	2.2	1.04E-002
Secreted phosphoprotein 1, Osteopontin	*SPP1*	3.3	5.99E-009	8.3	2.03E-007
Transmembrane 7 superfamily member 4	*TM7SF4*	4.1	6.27E-016	5.8	3.75E-002
Triggering receptor expressed on myeloid cells 2	*TREM2*	3.3	4.39E-016	8.6	2.00E-007

### Genes Induced by Cigarette Smoke in the Human and Mouse Lung

Genes are the main unit of comparison in most studies performed on clinical samples and animal models. Moreover, identifying the involvement of a gene in a given disease is a common research approach. Among the 17 genes significantly modulated by cigarette smoke in mice and humans, 10 were previously reported and investigated. These include MMP12, AHRR, SPP1, ALDH3A1, CYP1B1, GDF15, GSTA2, NQO1, PLA2G7, TREM2 and CLEC5A. The genes previously reported to be upregulated in smokers/COPD patients or cigarette smoke-exposed mice provide validation of our gene expression comparison between mice and humans. We also identified 6 new genes namely ACP5, ATP6V0D2, BHLHE41, NEK6, DCSTAMP and LCN2. These new genes associated with cigarette smoke exposure in both humans and mice may be of great interest for future research.

### Pathways Activated by Cigarette Smoke in the Human and Mouse Lung

**Figure 2:**
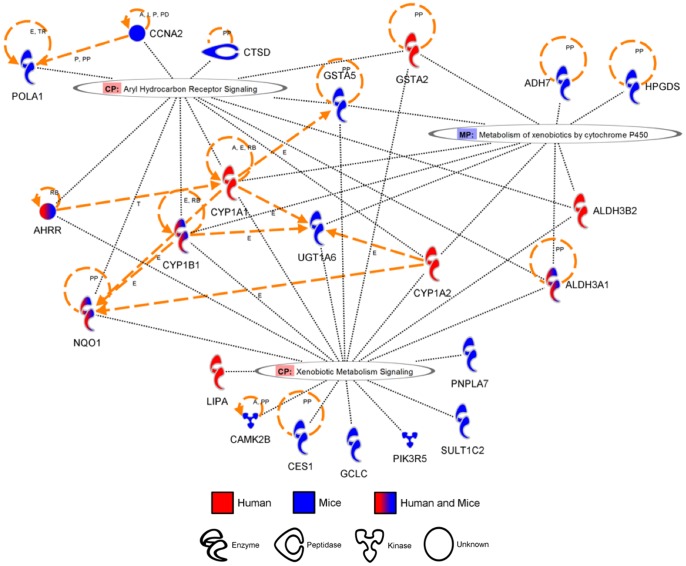
Genes associated to “Xenobiotics response/detoxification” pathways modulated by cigarette smoke exposure in humans and mice. Orthologue genes significantly upregulated in humans and/or mice (fold change >2) were used to predict canonical pathways activated by cigarette smoke exposure. Diagram generated using the Ingenuity Pathway Analysis Software.

**Figure 3:**
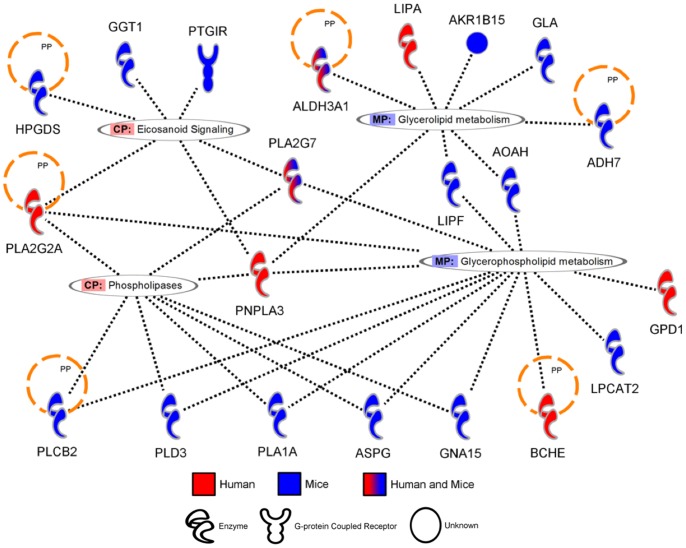
Genes associated to “Phospholipid metabolism/degradation” pathways modulated by cigarette smoke exposure in humans and mice. Orthologue genes significantly upregulated in humans and/or mice (fold change >2) were used to predict canonical pathways activated by cigarette smoke exposure. Diagram generated using the Ingenuity Pathway Analysis Software.

**Figure 4:**
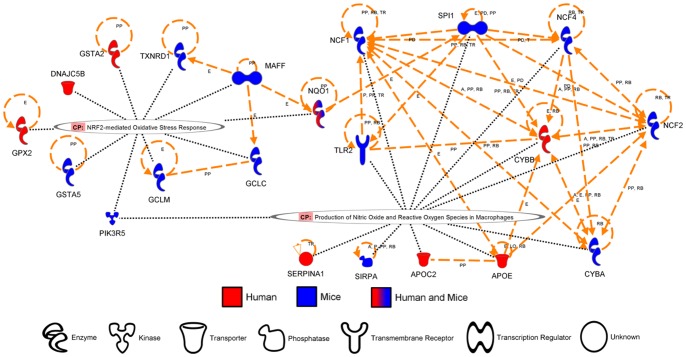
Genes associated to “Oxidative stress defense/generation” pathways modulated by cigarette smoke exposure in humans and mice. Orthologue genes significantly upregulated in humans and/or mice (fold change >2) were used to predict canonical pathways activated by cigarette smoke exposure. Diagram generated using the Ingenuity Pathway Analysis Software.

While the similarities between the two species are scarce at the gene level, it is possible that both species are initiating a similar response involving different genes. To identify pathways altered by cigarette smoke in humans and mice, canonical pathways from the Ingenuity Pathway Analysis Software were evaluated. The pathways activated by cigarette smoke in both mice and humans are presented in Table 3 and clustered around three main themes: xenobiotics response/detoxification (Figure 2), phospholipids metabolism/degradation (Figure 3), and oxidative stress defense/generation (Figure 4). Among the genes significantly modulated in both humans and mice, only AHRR, ALDH3A1, NQO1, GSTA2, CYP1B1 and PLA2G7 were found in the prediction of pathway activation. A majority of the genes involved in these pathways were not significantly activated in both humans and mice but were regrouped under the same pathways, suggesting that pathway activation signatures can be detected despite significant differences in the modulated genes. Interestingly, the majority of pathways associated with cigarette smoke exposure were activated only in humans (Table S1 in File S1) or in mice (Table S2 in File S1). In humans, this includes pathways clustering around IL-17 signaling, driven by the upregulation of CXCL1, IL-8 and CSF3, and atherosclerosis-associated pathways (i.e. atherosclerosis signaling, LXR/RXR activation, FXR\RXR activation), driven by the upregulation of apolipoprotein E (APOE) and C2 (APOC2). In mice, activated pathways were associated with innate and adaptive immunity (i.e. altered T cell and B cell signaling in rheumatoid arthritis, fMLP signaling in neutrophils), as well as glutathione and riboflavin metabolisms. Although the later pathways are interesting, no obvious pattern was observed in the pathways activated only in mice. In closing, we observed limited overlap between humans and mice on a gene per gene comparison. However, greater interspecies concordance was revealed in canonical signaling pathways.

**Table 3:** Common canonical pathways altered by cigarette smoke exposure in the human and mouse lungs.

Ingenuity Canonical Pathways	Species	p-value	Ratio	Genes
***Xenobiotic response/detoxification***				
**Aryl Hydrocarbon Receptor** **Signaling**	HUMANS	7.41E-006	5.67E-002	*AHRR, GSTA2, ALDH3B2, CYP1A1,* *CYP1A2, NQO1, ALDH3A1, CYP1B1*
	MICE	3.31E-003	5.76E-002	*AHRR, CTSD, CCNA2, POLA1, GSTA5,* *NQO1, ALDH3A1, CYP1B1*
**Metabolism of Xenobiotics by** **Cytochrome P450**	HUMANS	5.01E-005	6.90E-002	*GSTA2, ALDH3B2, CYP1A1, CYP1A2,* *ALDH3A1, CYP1B1*
	MICE	7.41E-003	6.45E-002	*ADH7, UGT1A6, GSTA5, HPGDS,* *ALDH3A1, CYP1B1*
**Xenobiotic Metabolism Signaling**	HUMANS	1.32E-004	3.45E-002	*AHRR, LIPA, GSTA2, ALDH3B2, CYP1A1,* *CYP1A2, NQO1, ALDH3A1, CYP1B1*
	MICE	3.39E-003	4.56E-002	*PNPLA7, AHRR, UGT1A6, Ces1g, GSTA5,* *Sult1c2, NQO1, PIK3R5, GCLC, ALDH3A1,* *CYP1B1, CAMK2B*
***Phospholipid metabolism/degradation***				
**Glycerophospholipid Metabolism**	HUMANS	2.40E-003	4.07E-002	*GPD1, PNPLA3, BCHE, PLA2G2A, PLA2G7*
	MICE	8.13E-003	5.65E-002	*PLCB2 (includes EG:18796), LPCAT2,* *PLD3, GNA15, ASPG, PLA1A, PLA2G7*
**Eicosanoid Signaling**	HUMANS	9.55E-003	5.17E-002	*PNPLA3, PLA2G2A, PLA2G7*
	MICE	2.09E-002	7.02E-002	*PTGIR, HPGDS, GGT1, PLA2G7*
**Phospholipid Degradation**	HUMANS	2.57E-002	3.57E-002	*PNPLA3, PLA2G2A, PLA2G7*
	MICE	4.37E-003	7.23E-002	*PLCB2 (includes EG:18796), PLD3,* *GNA15, ASPG, PLA1A, PLA2G7*
**Glycerolipid Metabolism**	HUMANS	3.16E-002	3.30E-002	*LIPA, PNPLA3, ALDH3A1*
	MICE	7.08E-003	6.52E-002	*AKR1B15, GLA, ADH7, LIPF,* *ALDH3A1, AOAH*
***Oxidative stress generation/defense***				
**NRF2-mediated Oxidative** **Stress Response**	HUMANS	4.57E-002	2.14E-002	*GSTA2, NQO1, GPX2, DNAJC5B*
	MICE	3.98E-002	3.89E-002	*GSTA5, NQO1, PIK3R5, GCLC,* *GCLM, MAFF, TXNRD1*
**Production of Nitric Oxide and** **Reactive Oxygen Species in** **Macrophages**	HUMANS	4.68E-002	2.16E-002	*APOE, CYBB, SERPINA1, APOC2*
	MICE	1.51E-002	4.52E-002	*TLR2, NCF1, CYBA, NCF2, PIK3R5,* *NCF4, SPI1 (includes EG:20375), SIRPA*
***Others***				
**Dendritic Cell Maturation**	HUMANS	2.75E-002	2.34E-002	*CD1A, CD1B, IL37, TREM2*
	MICE	1.05E-003	6.00E-002	*TLR2, FCGR2A, IL1RN, PIK3R5, FCER1G,* *CD86, LY75, FCGR2B, TREM2*
**Hematopoiesis from Pluripotent** **Stem Cells**	HUMANS	4.47E-003	5.45E-002	*IL8, LIF, CSF3*
	MICE	4.17E-002	6.25E-002	*KITLG, FCER1G, IGHA1*

### Biological Functions Affected by Cigarette Smoke in the Human and Mouse Lung

The lung response to cigarette smoke might not activate the same genes or trigger the same cellular and molecular pathways in both humans and mice but can still involve similar biological functions. In an effort to understand the global response of the human and mouse lung to cigarette smoke, genes were grouped under biological functions using the Ingenuity Pathway Analysis Software. Functions related to immune processes, metabolism and cell functions, diseases, tissue injury and repair, and organ development were observed (Table 4). Interestingly, most biological functions affected by cigarette smoke were observed in both humans and mice. This suggests that, despite differences in the molecular and cellular way humans and mice respond to cigarette smoke exposure, the global biological response is very similar.

**Table 4:** Common biological functions altered by cigarette smoke exposure in the human and mouse lungs.

Ingenuity BiologicalFunction	Species	p-value	Ingenuity BiologicalFunction	Species	p-value
***Immune system***					
**Inflammatory Response**	Human	1.19E-10-2.26E-02	**Cell-To-Cell Signaling and** **Interaction**	Human	1.15E-04-2.26E-02
	Mouse	1.35E-16-1.6E-02		Mouse	8.83E-11-1.67E-02
**Antigen Presentation**	Human	1.95E-07-2.26E-02	**Hypersensitivity Response**	Human	3.65E-03-2.26E-02
	Mouse	2.09E-08-1.45E-02		Mouse	8.89E-06-1.39E-02
**Cellular Movement**	Human	1.95E-07-2.26E-02	**Lymphoid Tissue Structure** **and Development**	Human	8.04E-03-8.04E-03
	Mouse	4.8E-13-1.62E-02		Mouse	3.74E-07-1.44E-02
**Hematological System** **Development and Function**	Human	1.95E-07-2.26E-02	**Antimicrobial Response**	Human	5.63E-03-5.63E-03
	Mouse	4.8E-13-1.62E-02		Mouse	7.24E-04-7.24E-04
**Immune Cell Trafficking**	Human	1.95E-07-2.26E-02	**Hematopoiesis**	Human	8.36E-04-2.26E-02
	Mouse	4.8E-13-1.62E-02		Mouse	3.74E-07-1.44E-02
**Cell-mediated Immune Response**	Human	3.31E-05-2.26E-02	**Inflammatory Disease**	Human	2.47E-04-2.26E-02
	Mouse	2.01E-05-1.44E-02		Mouse	2.55E-10-1.31E-02
***Metabolism and cellular maintenance***					
**Lipid Metabolism**	Human	4.18E-06-2.26E-02	**Cell Signaling**	Human	2.71E-04-2.71E-04
	Mouse	3.3E-05-1.39E-02		Mouse	1.82E-03-1.25E-02
**Energy Production**	Human	1.45E-05-1.51E-02	**Cellular Function and** **Maintenance**	Human	2.71E-04-2.26E-02
	Mouse	3.28E-03-3.28E-03		Mouse	5.33E-11-1.44E-02
**Vitamin and Mineral Metabolism**	Human	1.45E-05-2.26E-02	**Molecular Transport**	Human	2.71E-04-2.02E-02
	Mouse	1.82E-03-5.21E-03		Mouse	2.77E-04-1.39E-02
**Nucleic Acid Metabolism**	Human	2.3E-05-7.58E-03	**Gene Expression**	Human	1.51E-02-1.51E-02
	Mouse	1.99E-03-1.99E-03		Mouse	1.19E-02-1.19E-02
**Drug Metabolism**	Human	4.22E-07-2.26E-02	**Cell Morphology**	Human	4.76E-03-1.51E-02
	Mouse	5.6E-07-2.77E-04		Mouse	9.66E-04-1.26E-02
**Carbohydrate Metabolism**	Human	5.6E-04-2.26E-02	**Cellular Assembly and** **Organization**	Human	3.57E-03-1.51E-02
	Mouse	4.85E-03-1.18E-02		Mouse	4.36E-04-1.39E-02
**Small Molecule Biochemistry**	Human	4.22E-07-2.26E-02			
	Mouse	3.3E-05-1.39E-02			
***Diseases***					
**Cancer**	Human	2.44E-05-2.26E-02	**Hepatic System Disease**	Human	1.63E-03-2.26E-02
	Mouse	1.41E-06-1.56E-02		Mouse	3.28E-03-1.54E-02
**Respiratory Disease**	Human	2.44E-05-2.15E-02	**Ophthalmic Disease**	Human	4.18E-03-2.26E-02
	Mouse	2.72E-04-1.64E-02		Mouse	1.01E-03-1.01E-03
**Cardiovascular Disease**	Human	2.66E-04-1.27E-02	**Tumor Morphology**	Human	7.13E-03-2.26E-02
	Mouse	4.67E-05-1.61E-04		Mouse	1.01E-03-5.63E-03
**Metabolic Disease**	Human	2.66E-04-2.26E-02	**Endocrine System Disorders**	Human	8.99E-03-1.44E-02
	Mouse	1.25E-02-1.25E-02		Mouse	8.49E-05-1.25E-02
**Connective Tissue Disorders**	Human	3.86E-04-2.26E-02	**Infectious Disease**	Human	1.4E-03-2.26E-02
	Mouse	2.55E-10-1.18E-02		Mouse	2.8E-09-1.32E-02
**Skeletal and Muscular Disorders**	Human	3.86E-04-2.26E-02	**Genetic Disorder**	Human	4.04E-05-2.26E-02
	Mouse	2.55E-10-1.53E-02		Mouse	9.79E-05-1.53E-02
**Renal and Urological Disease**	Human	6.14E-04-2.26E-02	**Hematological Disease**	Human	5.68E-05-2.17E-02
	Mouse	1.68E-03-1.68E-03		Mouse	3.4E-04-1.06E-02
**Gastrointestinal Disease**	Human	6.67E-04-2.26E-02	**Dermatological Diseases and** **Conditions**	Human	1.45E-04-2.26E-02
	Mouse	1.01E-03-1.54E-02		Mouse	4.82E-04-1.44E-02
**Neurological Disease**	Human	1.21E-03-2.26E-02	**Immunological Disease**	Human	1.7E-04-2.26E-02
	Mouse	4.94E-04-1.53E-02		Mouse	2.19E-09-1.67E-02
***Cell injury and repair***					
**Cell Death**	Human	1.31E-03-2.26E-02	**Organismal Survival**	Human	1.24E-02-1.24E-02
	Mouse	8.56E-06-1.63E-02		Mouse	5.49E-03-1.56E-02
**Cellular Compromise**	Human	2.13E-03-2.26E-02	**Free Radical Scavenging**	Human	5.86E-04-9.62E-04
	Mouse	4.41E-07-1.12E-02		Mouse	6.79E-04-1.5E-02
**Cell Cycle**	Human	7.58E-03-2.26E-02	**Cellular Growth and** **Proliferation**	Human	1.66E-06-2.26E-02
	Mouse	3.73E-04-1.67E-02		Mouse	9.66E-08-1.44E-02
**DNA Replication,** **Recombination, and Repair**	Human	1.51E-02-1.51E-02	**Organismal Injury and** **Abnormalities**	Human	2.15E-03-2.15E-02
	Mouse	4.36E-04-1.67E-02		Mouse	4.46E-05-1.64E-02
***Development***					
**Skeletal and Muscular System** **Development and Function**	Human	1.66E-06-1.51E-02	**Hair and Skin Development** **and Function**	Human	1.34E-03-1.51E-02
	Mouse	1.72E-06-1.67E-02		Mouse	3.5E-03-3.5E-03
**Endocrine System Development** **and Function**	Human	1.68E-06-1.51E-02	**Nervous System Development** **and Function**	Human	1.63E-03-1.51E-02
	Mouse	1.99E-03-1.21E-02		Mouse	3.4E-04-1.39E-02
**Tissue Development**	Human	1.15E-04-2.26E-02	**Organ Development**	Human	7.58E-03-7.58E-03
	Mouse	1.72E-06-1.45E-02		Mouse	1.83E-03-1.31E-02
**Developmental Disorder**	Human	1.7E-04-2.26E-02	**Organ Morphology**	Human	7.58E-03-7.58E-03
	Mouse	9.79E-05-3.28E-03		Mouse	6.06E-05-1.39E-02
**Tissue Morphology**	Human	3.38E-04-2.26E-02	**Organismal Development**	Human	7.58E-03-1.51E-02
	Mouse	2.92E-09-1.67E-02		Mouse	3.4E-04-1.31E-02
**Renal and Urological System** **Development and Function**	Human	5.39E-04-1.51E-02	**Visual System Development** **and Function**	Human	7.58E-03-2.26E-02
	Mouse	1.01E-03-1.01E-03		Mouse	7.87E-03-1.31E-02
**Cardiovascular System** **Development and Function**	Human	1.34E-03-2.26E-02	**Organismal Functions**	Human	1.41E-02-1.41E-02
	Mouse	5.92E-05-1.6E-02		Mouse	3.28E-03-4.03E-03
**Embryonic Development**	Human	1.34E-03-1.51E-02	**Connective Tissue Development** **and Function**	Human	1.51E-02-2.26E-02
	Mouse	3.4E-04-1.99E-03		Mouse	1.72E-06-1.67E-02

## Discussion

The present study aimed at comparing the human and mouse lung gene expression signature to cigarette smoke exposure. To do so, we compared two genome-wide expression experiments, one in human lungs of non-smokers and smokers and the other one in lungs of mice exposed to room air or cigarette smoke. This interspecies gene expression comparison allowed us to identify 6 new genes modulated by cigarette smoke. Moreover, we confirmed the activation of pathways linked to xenobiotics detoxification and oxidative stress defense/generation. We also identified a new response conserved among mice and humans in the activation of pathways involved in phospholipid metabolism and degradation. Finally, we found that biological functions affected by cigarette smoke exposure are largely similar in human and mouse lung.

### Genes Previously Associated to Cigarette Smoke Exposure

Our study confirmed the impact of cigarette smoke on smoking-induced genes previously described in humans and mice. The role of MMP12 [9], [10], SPP1 [11], AHRR [12], [13] and CYP1B1 [13], [14] in cigarette smoke-related lung diseases have been well described and studied. Other genes have been shown to be induced by cigarette smoke in humans or mice but only marginally studied so far. NQO1 is likely associated with the detoxification of aldehydes [15] and CLEC5A and TREM2 with myeloid cell activation [16], [17]. GSTA2 is likely involved in the antioxidant response [18]. The roles played by “growth and differentiation factor 15″ (GDF15) and “lipoprotein-associated phospholipase A2” (Lp-PLA2, encoded by the gene PLA2G7) however, are less intuitive. GDF15 (a.k.a. macrophage inhibitory cytokine-1 [MIC-1]) is a divergent member of the TGF-β superfamily [19] and can be secreted by activated macrophages [20]. GDF15 is overexpressed in many tumor types and following tissue injury, including lung injury [21]. It has been linked to the development of atherosclerosis as well as the regulation of adiposity in mice [22]–[24]. *Wu et al*. showed that GDF15 expression is increased in the lung of COPD patients as well as in human lung epithelial cells exposed to cigarette smoke [25]. They also found GDF15 to be an inducer of MUC5A [25]. Lp-PLA2 is an extracellular phospholipase involved in the inactivation of the “Platelet-Activating Factor” (PAF) into lyso-PAF and in the cleavage of oxidized phospholipids [26]. It is produced by inflammatory cells and found in low-density lipoproteins (LDLs) (a.k.a LDL-PLA2) [26]. Increased serum Lp-PLA2 activity is observed in patients and mice with atherosclerosis [27], [28], its role in the disease progression being debated. Interestingly, increased serum Lp-PLA2 activity is also observed in smokers when compared to nonsmokers [29]. The upregulation of PLA2G7 by cigarette smoke may therefore reflect the activation of modified phospholipids-neutralizing mechanisms within the lung environment. Although the functions of GDF15 and Lp-PLA2 in the lung homeostasis are still unknown, their association to cancer, atherosclerosis, tissue injury or lipid metabolism makes them important research targets in the context of cigarette smoke-related lung diseases.

### Unstudied Genes Associated with Cigarette Smoke Exposure

The genes ACP5, ATP6V0D2, BHLHE41, NEK6, DCSTAMP and LCN2 were found to be associated to cigarette smoke exposure but to the best of our knowledge, their roles in smoking-related diseases were never studied. Class E basic helix-loop-helix protein 41 (BHLHE41) is a transcription factor that has an impact on a variety of cellular functions. Its expression can also be induced by many stimuli such a growth factors [30]. NimA-related protein kinase 6 (NEK6) plays an important role in the mitotic cycle [31]. It can phosphorylate STAT3 and histones 1 and 3 and its suppression leads to cell apoptosis [31]. It is also involved in G2/M phase cell cycle arrest induced by DNA damage [31]. The gene LCN2 encodes the enzyme “neutrophil gelatinase-associated lipocalin” (NGAL). NGAL binds to bacterial iron-chelating siderophores to prevent iron uptake and impair bacterial growth [32]. ATPase, H^+^ transporting, lysosomal 38kDa, V0 subunit d2 *(*ATP6V0D2*)* is a member of the H^+^-ATPase family and is responsible for the acidification of the extracellular environment by osteoclasts [33]. Its role in bone degradation is well described [33], [34]. *Atp6v0d2* null mice exhibit osteopetrosis also called the “stone bone” disease [35], [36]. Tartrate-Resistant Acid Phosphatase 5, (ACP5) or Tartrate-Resistant Acid ATPase (TRAP) is produced and secreted by activated macrophages including osteoclasts and can catalyze the generation of reactive oxygen species (ROS), mediate iron transport and dephosphorylate osteopontin [37]. *Acp5* knockout mice have mild osteoporosis [38]. DCSTAMP that encodes the trans-membrane protein DC-STAMP is expressed by dendritic cells and macrophages and is required for the cell-cell fusion required for the formation of foreign-body giant cells and osteoclasts [39].

The involvement of MMP12, AHRR, SPP1, GSTA2, ALDH3A1, CYP1B1, NQO1, TREM2 and CLEC5A in the lung response to cigarette smoke can be associated to immune or detoxification responses. However, the reasons for ACP5, ATP6V0D2, BHLHE41, NEK6, TM7SF4, LCN2, GDF15 and PLA2G7 upregulation are less intuitive. Interestingly, the genes ATP6V0D2, ACP5, DCSTAMP and SPP1 have been mainly studied for their role in bone homeostasis and are expressed by bone-resorbing osteoclasts. Further studies on the involvement of those genes in cigarette smoke-associated disease are required.

### Canonical Pathways Induced by Cigarette Smoke

We found that the three major clusters of cellular pathways activated in the lung of mice and humans exposed to cigarette smoke were the response to xenobiotics/aryl hydrocarbon receptor activation, oxidative stress defense/generation and lipid/phospholipid degradation. The presence of xenobiotic compounds in cigarette smoke such as poly aromatic hydrocarbons (PAH) and the associated detoxification response involving the aryl hydrocarbon receptor (AHR) and members of the cytochrome p450 family has been well described [12], [40], [41]. However, the long-term effects of xenobiotics exposure as well as AHR activation on the immune system and lung homeostasis are not as well understood. Activation of the AHR pathway has both pro- and anti-inflammatory properties [42]. *Ahr*-deficient mice exhibit a more robust pulmonary inflammation than their wildtype counter-parts when exposed to cigarette smoke [12]. AHR activation has been link to cancer [41] and is also believed to promote the development of a Th17 response [42].

The role of oxidative stress is one of the oldest research ground in the field of cigarette smoke exposure. Cigarette smoke contains more than 10^15^ reactive oxygen species (ROS) per puff as well as reactive aldehydes and heavy metals [43]. The effects of oxidative stress on the immune system and lung injury are extensively studied. It is therefore not surprising to see the activation of the antioxidant response by cigarette smoke conserved between mice and humans.

Pathways involved in phospholipid metabolism and degradation were activated in both the human and mouse lungs. Three genes in humans and four genes in mice were coding for phospholipases. Phospholipases are enzymes that hydrolyze phospholipids into fatty acids and other compounds, releasing modified fatty acids such as arachidonic acid and second messengers that will be used for eicosanoid synthesis or activate intracellular signaling [44]. These mediators can activate immune cells and promote inflammation [44]. In the lung, phospholipids can be found in cellular membranes, in the surfactant as well as in lipoproteins. Activation of these pathways suggests a high phospholipid turnover that could be a result of direct damage made by cigarette smoke. Signs of damaged phospholipids have been found in both humans and mice exposed to cigarette smoke in the presence of lipid peroxidation products and oxidized phospholipids [45], [46]. The conserved activation of pathways involved in phospholipids metabolism and degradation by cigarette smoke in both the human and mouse lung is a novel finding, suggesting its importance in the response to cigarette smoke.

### Biological Functions Induced by Cigarette Smoke

Cigarette smoke exposure in humans and mice were both reflected in the activation of biological functions related to inflammation, metabolism and cell maintenance, injury and repair, diseases and development. The role of inflammation, injury and repair are the focus of most of the research done on cigarette smoke exposure. However, cigarette smoke seems to affect genes involved in a broad range of functions related to cell maintenance and metabolism. More specifically, the metabolism of major cellular constituents such as lipids, carbohydrates, vitamins and nucleic acids is affected. Therefore, it is not surprising that cigarette smoke has so many systemic effects. In fact, many genes and functions related to non-pulmonary diseases and to the development of other organs are affected within the lungs. It is tempting to speculate that genes affected by cigarette smoke within the lungs may also be affected in other tissues/organs. Finally, the near complete overlap of biological functions affected by cigarette smoke between humans and mice validates the use of the animal model to explore the mechanisms leading to their activation.

An inherent limitation of this study was the use of a single inbred mouse strain. It is plausible that the regulation of some genes may only be significant in this strain. Further studies are required to compare gene expression profiles across different inbred mouse strains. In addition, the majority of the clinical samples were obtained from subjects undergoing surgery for lung cancer. Although, we collected non-tumor specimens as distant as possible from the tumor, cancer might still interfere with the expression of some genes and pathways.

In the present study, we found that similarities in the transcriptomic response of the lungs between humans and mice exposed to cigarette smoke reside more at the pathway and functional levels rather than the single gene level. Beyond the basic research findings, this study provides empirical data to support that limiting analysis to single genes in animal models may, in many cases, compromise the translational potential to the clinic. While mice and humans show great similarities in the response to many different stimuli, it is likely that they activate different genes, or molecular languages, to achieve the same goal. Therefore, rather than studying the involvement of specific genes, efforts should be deployed to understand the role played by specific pathways or functions. This more integrative approach in mouse would likely improve the translational potential of new findings derived from this unsurpassed pre-clinical model to study human biology and disease.

## Supporting Information

File S1 **Supporting figures and tables. Figure S1. Genes in common (orthologue) between the human and mouse expression microarrays.** Human (20,025 genes) and mouse (28,038 genes) microarrays were compared on the basis of gene names to identify orthologue genes with probes present in both sets. **Figure S2. Enrichment plot for genes modulated by cigarette smoke in mice.** Genes modulated by cigarette smoke in mice were tested for enrichment against the genes modulated by cigarette smoke in humans pre-ranked according to their fold change. **Table S1. Canonical pathways altered by cigarette smoke in the human lung only. Table S2. Canonical pathways altered by cigarette smoke in the mouse lung only.**
(DOC)
