# Supplementary material for: Impact of Cigarette Smoke on the Human and Mouse Lungs: A Gene-Expression Comparison Study
Source: PLoS One. 2014 Mar 24;9(3):e92498. doi: 10.1371/journal.pone.0092498 (PMC3963906; doi:10.1371/journal.pone.0092498)
Supplement: File S1 — Supporting figures and tables. Figure S1. Genes in common (orthologue) between the human and mouse expression microarrays. Human (20,025 genes) and mouse (28,038 genes) microarrays were compared on the basis of gene names to identify orthologue genes with probes present in both sets. Figure S2. Enrichment plot for genes modulated by cigarette smoke in mice. Genes modulated by cigarette smoke in mice were tested for enrichment against the genes modulated by cigarette smoke in humans pre-ranked according to their fold change. Table S1. Canonical pathways altered by cigarette smoke in the human lung only. Table S2. Canonical pathways altered by cigarette smoke in the mouse lung only. (DOC) [file pone.0092498.s001.doc]

**SUPPLEMENTARY INFORMATION DOCUMENT**

**Impact of cigarette smoke on the human and mouse lungs: a gene-expression comparison study**

**FIGURE S1**


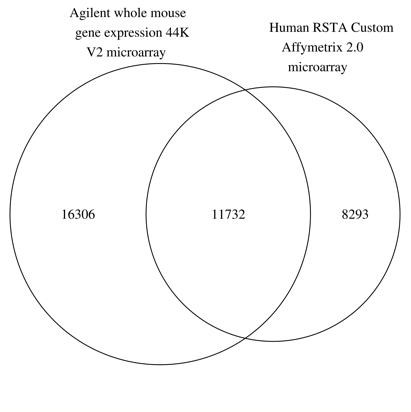


**FIGURE S2**

**
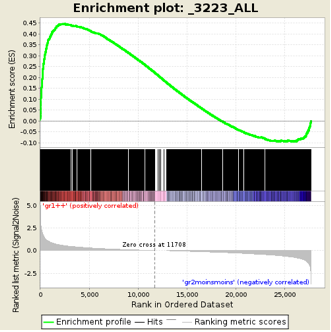
**

**TABLE S1.** Canonical pathways altered by cigarette smoke in the human lung only

**TABLE S2.** Canonical pathways altered by cigarette smoke in the mouse lung only
